# Population genetic study of 34 X-Chromosome markers in 5 main ethnic groups of China

**DOI:** 10.1038/srep17711

**Published:** 2015-12-04

**Authors:** Suhua Zhang, Yingnan Bian, Li Li, Kuan Sun, Zheng wang, Qi Zhao, Lagabaiyila Zha, Jifeng Cai, Yuzhen Gao, Chaoneng Ji, Chengtao Li

**Affiliations:** 1 Shanghai Key Laboratory of Forensic Medicine, Institute of Forensic Sciences, Ministry of Justice, P.R. China, Shanghai 200063, P.R. China; 2 State Key Laboratory of Genetic Engineering, Institute of Genetics, School of Life Sciences, Fudan University, Shanghai 200433, P.R. China; 3 Institute of Forensic Medicine, West China School of Basic Science and Forensic Medicine, Sichuan University, Chengdu 610041, P.R.China; 4 Department of Forensic Science, School of Basic Medical Sciences, Central South University, Changsha 410013, P.R. China; 5 Department of Forensic Medicine, Medical College of Soochow University, Suzhou 215123, P.R. China

## Abstract

As a multi-ethnic country, China has some indigenous population groups which vary in culture and social customs, perhaps as a result of geographic isolation and different traditions. However, upon close interactions and intermarriage, admixture of different gene pools among these ethnic groups may occur. In order to gain more insight on the genetic background of X-Chromosome from these ethnic groups, a set of X-markers (18 X-STRs and 16 X-Indels) was genotyped in 5 main ethnic groups of China (HAN, HUI, Uygur, Mongolian, Tibetan). Twenty-three private alleles were detected in HAN, Uygur, Tibetan and Mongolian. Significant differences (p < 0.0001) were all observed for the 3 parameters of heterozygosity (Ho, He and UHe) among the 5 ethnic groups. Highest values of Nei genetic distance were always observed at HUI-Uygur pairwise when analyzed with X-STRs or X-Indels separately and combined. Phylogenetic tree and PCA analyses revealed a clear pattern of population differentiation of HUI and Uygur. However, the HAN, Tibetan and Mongolian ethnic groups were closely clustered. Eighteen X-Indels exhibited in general congruent phylogenetic signal and similar cluster among the 5 ethnic groups compared with 16 X-STRs. Aforementioned results proved the genetic polymorphism and potential of the 34 X-markers in the 5 ethnic groups.

Identification of the genetic information of populations is an important task for which different types of genetic markers can be used, including autosomal^1^,^2^, mtDNA^2^,^3^, Y-Chromosome^2^,^3^,^4^ and X-Chromosome^5^,^6^,^7^. The use of autosomal markers is a typical strategy to disclose population patterns, as these markers account for both paternal and maternal inheritance. Nevertheless, X-Chromosome is gaining significant importance in population and forensic genetic studies due to the special transmission property^5^,^6^,^7^. The X-Chromosome is transmitted between both sexes in each generation, telling a different story from uniparental genomes. Moreover, its effective population size is reduced in relation to autosomes, making it more sensitive to the effects of population substructure and genetic drift^6^,^7^. And X-Chromosome markers show higher efficiency parameters than autosomes in special kinship investigations involving mainly female offspring, making them suitable for forensic application^6^,^7^. Thus, growing number of scientists is becoming interested in X-Chromosome research, using X markers (X-STRs, X-SNPs and more recently X-Indels) for studying the genetic structure of human populations, ancestry proportions in admixed populations as well as for forensic investigations^5^,^6^,^7^,^8^,^9^. Genetic markers of STRs or SNPs are predominantly applied to dissect patterns of genetic variation for human genetic studies. Indels represent another type of DNA variation that is increasingly studied and applied in genetics^6^,^8^,^9^. However, studies of X-Chromosome markers in Chinese population have only been related to genetic parameters, home-system development or evaluation. As a large united multi-ethnic state, composed of 56 ethnic groups, studies on the genetic background of China are necessary. This study was focused on 5 main ethnic groups, including HAN and 4 main minority ethnic groups (HUI, Uygur, Mongolian and Tibetan). HAN Chinese account for almost 92% of China’s population, and make up roughly 20% of the international population, making it the world’s largest ethnic group. Here, HAN population in Shanghai City was chosen to be studied since the highest proportion of Shanghai’s residents are members of China’s vast floating population. Additionally, the 4 minority ethnic groups are mainly located in Northwest of China. All of them have populations of more than 5 million according to 2010 national data and are regarded as typical examples of Chinese ethnic minorities. The detail location of each ethnic group is depicted in Supplementary Fig. S1.

Some Chinese investigators have examined population differentiation and admixture patterns for Chinese ethnic groups and some Central Asian populations with mtDNA or Y chromosomes^10^,^11^. Previous studies demonstrated that high genetic differentiation exists among Chinese ethnic groups and that the gene flow and genetic admixture are very complex. Addressing major issues in the field of human genetics requires multiple types of genetic markers, various analytical methods and statistical models. In the present study, a set of 34 X- chromosomal markers (16 X-STRs and 18 X-Indels) was evaluated in the 5 main ethnic groups of China. The 4 minority ethnic groups along the Silk Road in the Northwest of China display clear differences in culture and social customs, perhaps as a result of geographic isolation and different traditions. However, extensive trade and interactions probably facilitated the admixture of different gene pools between these ethnic groups over the last two millennia. The aim was to obtain detailed genetic information of the 34 X-Chromosome markers and to improve the current knowledge on the genetic background of the 5 ethnic groups of China.

## Results and Discussion

### Genetic parameters of the 34 X-Chromosomal markers

34 X-Chromosomal markers (16 X-STRs and 18 X-Indels) of 500 samples from 5 main ethnic groups of China were genotyped for 100 individuals in each ethnic group (50 females and 50 males). The genotyping profiles of control DNA 9947A (0.5 ng) amplified with Panel I (16 X-STRs) and Panel II (18 X-Indels) are listed as Supplementary Fig. S2-A and Fig. S2-B, respectively. The amplification system and cycling conditions of Panel I and II are listed in Supplementary Table S1. Apart from using different primer mix, the other PCR components and PCR conditions are same for the Panel I and II.

Since the 34 studied markers were located on X-Chromosome, the allelic frequency of each marker in each population was calculated in both male and female samples. As no significant differences were observed (*p* > 0.05), male and female samples were pooled for each ethnic group and used for further investigation. Allelic frequencies of the 34 X-Chromosome markers are listed in Supplementary Table S2. The data varied for each population, enriching the database of the X-Chromosome markers. No deviations from Hardy–Weinberg equilibrium were observed in any of the ethnic groups or markers after the correction for multiple tests. In total 131 alleles were detected among the 16 X-STRs in the 5 ethnic groups. DXS101 was detected with the 17 alleles at the maximum, while DXS9902 was only detected with 5 alleles among the 5 ethnic groups. Twenty-three private alleles (alleles that are found only in a single population) were detected of 4 ethnic groups (Table 1). No private alleles were detected in the HUI population. Up to 10 private alleles were detected in the Tibetan population. Some alleles (allele 17 of DXS101, allele 28 of DXS6809, allele 13 of DXS7423, allele 19 of DXS7133 and allele 10 of DXS6810) were detected with frequencies of 0.08 in a certain population (Table 1). Private alleles have proven to be informative for diverse types of population-genetic studies, in such areas as molecular ecology and conservation genetics^12^ and human evolutionary genetics^13^. The information on private alleles may become enriched if a broader collection of ethnic groups were investigated.

Based on the genotyping information, the Observed Heterozygosity (Ho), Expected Heterozygosity (He) and Unbiased Expected Heterozygosity (UHe) of the 34 markers in the 5 ethnic groups were calculated with the GENALEX 6.3 software (Supplementary Table S3). Heterozygosity can be used as a measure of a population’s capacity to respond to natural selection immediately after a bottleneck. Allelic diversity, on the other hand, determines a population’s ability to respond to long-term selection over many generations. For the 16 X-STRs, the mean Ho values were 0.6660 ± 0.1222, 0.6423 ± 0.0916, 0.7050 ± 0.1111, 0.6401 ± 0.1266 and 0.6732 ± 0.1158 and the mean He values were 0.6685 ± 0.1105, 0.6262 ± 0.1186, 0.7092 ± 0.0775, 0.6572 ± 0.1119 and 0.6798 ± 0.1007 for HAN, HUI, Uygur, Mongolian and Tibetan, respectively. As seen in Supplementary Fig. S3-A, the STR of DXS7132 has the highest standard deviation (SD) with 0.66956 ± 0.1190 for Ho values while DXS7423 enjoy the lowest SD with 0.50596 ± 0.030 among the five ethnic groups. For the 18 X-Indels, the mean Ho values were 0.4365 ± 0.1367, 0.3594 ± 0.1031, 0.3300 ± 0.1096, 0.3421 ± 0.1129 and 0.3618 ± 0.1222 and the mean He values were 0.4006 ± 0.1002, 0.3867 ± 0.1017, 0.3881 ± 0.1091, 0.3699 ± 0.1107 and 0.3751 ± 0.1212 for HAN, HUI, Uygur, Mongolian and Tibetan, respectively. Indel of rs3215490 has the highest standard deviation (SD) with 0.3853 ± 0.1860 for Ho values and 0.3502 ± 0.1281 for UHe values among the five ethnic groups (Supplementary Fig. S3-B). For the Ho values, Indel of rs25581 enjoy the highest SD (0.3506 ± 0.1231) while rs3215490 enjoy the second highest SD (0.3424 ± 0.1227). Significant differences (*p* < 0.0001) were observed among the 5 ethnic groups with each kind of heterozygosity. Population heterozygosity varied greatly from locus to locus as seen in Supplementary Table S3 and Supplementary Fig. S3-A,B, which results from evolutionary forces such as mutation, selection, and random genetic drift. In addition, since STRs can have numerous alleles compared to bi-allelic markers, the heterozygosity of STRs is expectedly much higher than Indels. Among the 16 X-STRs, the minimum value of Ho was 0.4016 at DXS7133 (HUI ethnic group) and the maximum value of Ho was 0.8929 at DXS6809 (Mongolian ethnic group); among the 18 X-InDels, the minimum value of Ho was 0.1667 at rs72417152 (Tibetan ethnic group) and maximum value of Ho was 0.7500 at rs45449991 (Han ethnic group). The heterozygosity information described above suggests that it is feasible to study the genetic background, population differentiation and admixture with the 34 markers utilized here.

Linkage Disequilibrium (LD) testing of the 34 X-Chromosome markers was explored with Arlequin 3.1 software. No LD was observed neither among the 16 X-STRs nor the 18 X-Indels. Thus the cumulative power of exclusion (the probability to exclude the unrelated male from putative father in trios involving daughters, as well as in father–daughter duos without information about the maternal genotype with the system) of the 16 X-STRs ranged from 0.999985835 (HAN ethnic group) to 0.99999846 (Uygur ethnic group) and the cumulative power of exclusion of the 16 X-Indels ranged from 0.999045398 (HUI ethnic group) to 0.99997081 (HAN ethnic group) among the 5 ethnic groups.

### Population pairwise differences and principal component analysis

A population comparison was made among all testing samples. Ten pairwise ethnic groups were obtained among the 5 ethnic groups. Upon analysis with the two different types of X-Chromosome markers separately and combined, Nei genetic distances of the 10 pairwise ethnic groups were listed in Table 2. Quantification of genetic distances between ethnic groups is essential in many genetic research studies^14^,^15^. Several equations have been proposed for estimation of the genetic distance between ethnic groups using the frequency data. Nei’s D has been the most widely used genetic distance measure in different research programs^14^,^15^. The results presented in Table 2 indicated that the Nei genetic distances between HUI and Uygur are always the highest, regardless of markers used for analysis. HUI and Uygur are the two major Muslim ethnic groups located in different areas (see Supplementary Fig. S1). Uygurs, known to be an admixture of Eastern Asian and European populations^16^, mostly reside in the Xinjiang Uyghur Autonomous Region. In this region, all residents are aboriginals except for the Han ethnic group, which has migrated to the region from Central China since the 1950s. The Uyghur ethnic group has been in frequent contact with both eastern and western populations since the 3rd Century B.C. Hui are concentrated within the Ningxia Hui Autonomous Region. The origin of Hui is diverse, and it is believed that they include individuals whose ancestors originated in pre-Islamic times, from Central Asia, Iran, and the Middle East^16^. Previous papers considered geography to be the main factor for ethnic group differences and argued that language exerted a secondary but detectable effect^5^,^8^,^9^,^10^,^16^. According to Zhang Z *et al.*^16^, genetic diversity of 13 populations residing in Northwest China analysed with auto-STRs demonstrate that Uygur living in northern China in Xinjiang Uighur Autonomous Region are quite distant from other populations living in Qinghai and Gansu and from the subpopulations of the Han, Hui and Mongol. Our results here confirmed the finding that Uygur and HUI have quite different genetic background.

**Figure 1:**
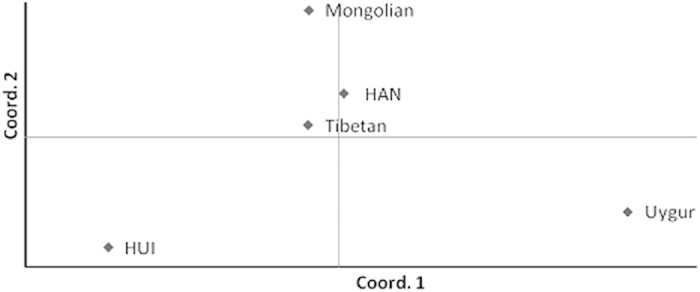
PCA based on Nei genetic distance matrix analyzed with 16 X-STRs and 18 X-Indels among the 5 ethnic groups.

Comparison of the data from three groups (Table 2), the group B (18 X-Indels) expectedly provided less information about the genetic diversity than the other two groups. However, even the rather limited number of biallelic loci (Indels) here can provide useful information about the general level of genetic diversity. To improve the visualization of the results, the pairwise genetic distances were represented in MDS plots with principal component analysis (PCA). PCA extracted several PCs as new variables by the dimension reduction method, which can be used to determine features and basic characteristics for differentiation among ethnic groups. PCA was performed with the Nei genetic distance matrix using the GENALEX 6.3 software. The results analysed for 16 X-STRs or 18 X-Indels are shown in Supplementary Fig. S4-A and Supplementary Fig. S4-B, respectively. As illustrated in Supplementary Fig. S4-A, the first two principal components defined 77.06% of the total variance, with the first and second component accounted for 50.90% and 26.12%, respectively. According to Supplementary Fig. S4-B, the first two principal components defined 79.24% of the total variance, with the first and second component accounted for 57.02% and 22.22%, respectively. In both figures, the Uygur distributed in the right-middle part whereas the HUI group clustered in the lower left quadrant of the plots. The other three groups were clustered in the upper-middle of the plots. The distributions of HAN and Tibetan in Supplementary Fig. S4-A,B indicated that 16 X-STRs have higher discrimination power to distinguish the two ethnic groups. We further combined these 34 markers together to perform the PCA analysis and the results are shown in Fig. 1. The first two principal components defined 75.88% of the total variance, with the first and second component accounting for 45.36% and 30.52%, respectively (Fig. 1), and the general pattern is similar to Supplementary Fig. S4-A,B.

### Phylogenetic analyses

**Figure 2:**
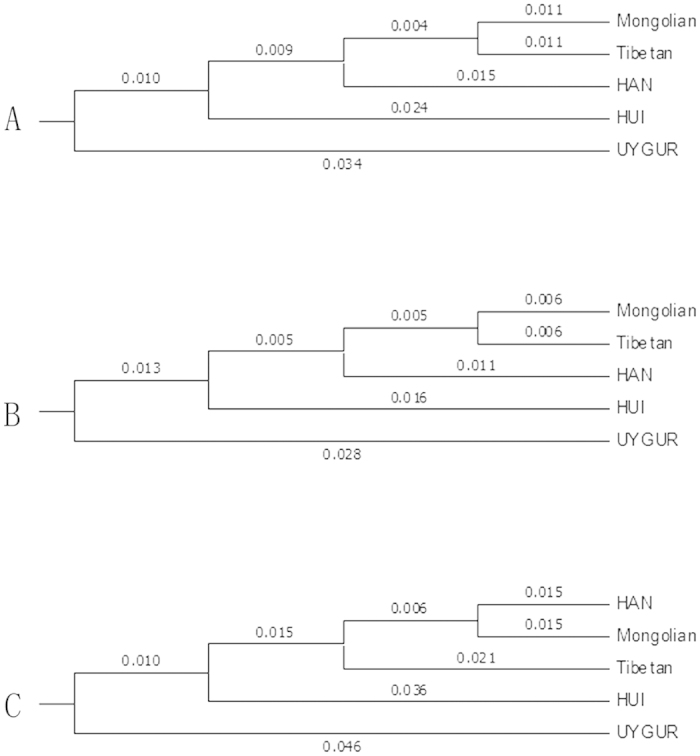
UPGMA phylograms for (**A**) STRs data, (**B**) Indels data, or (**C**) the combined STRs and Indels datasets. The trees are drawn to scale, with branch lengths in the same units as those of the evolutionary distances used to infer the phylogenetic tree.

Complementary phylogenetic trees, constructed from specific genetic distances, can easily deduce the evolutionary relationships and origins of different populations^17^,^18^. The Unweighted Pair Group Method with Arithmetic mean (UPGMA) method was applied for the phylogenetic reconstruction. Figure 2 shows the phylogenetic trees established with the genetic distance matrix obtained from STRs/Indels or makers combined among the 5 ethnic groups. The results were similar to the Supplementary Fig. S4-A,B and Fig. 1. Although Indels are less polymorphic compared to STRs, they provided general congruent phylogenetic signal and resulted in the same clustering of the 5 ethnic groups (Fig. 2A VS Fig. 2B). Indel polymorphisms produced by the insertion/deletion of one or more nucleotides allow for capillary genotyping, have a low mutation rate and a low recurrence, can present significant differences in the allele frequencies among geographically distant ethnic groups, and thus they have become increasingly popular markers for various population genetics applications. Indels represent approximately 20% of all polymorphisms in the human genome^19^,^20^. Also, ascertainment bias is a further issue that needs to be considered when using Indel markers for population genetic analyses as it may introduce a systematic bias in estimates of variation within and between ethnic groups^21^.

When the Indels data were combined with the STRs data for phylogenetic analyses, some small changes in population groupings and slight increases in bootstrap support for some nodes of the UPGMA phylogram were observed (Fig. 2C). The relationships among HAN, Tibetan and Mongolian ethnic groups were not so clear when analysed only with the 34 X-Chromosomal markers. The HAN population in this study was chosen from Shanghai, which is a vast floating place with population changes and intermarriage. Furthermore, in Chinese history, thousands of HAN people moved to the Tibetan and Mongolian capital to “support the advancement of the region”. Therefore, intermarriage between individuals from these ethnic groups could be a factor. All those findings reveal that geographic isolation and interactions play significant roles in population differentiation. Thus, in future studies, it would be interesting to increase the number of markers and ethnic groups analysed in order to assess more data from the main ethnic groups of the Chinese population.

## Conclusions

16 X-STRs and 18 X-Indels were detected with same PCR components (except for the primer mixes) and PCR conditions in 5 main ethnic groups of China. The genetic data varied for each population and enriched the database of the X-Chromosome markers. Twenty-three private alleles were detected among 4 ethnic groups (except HUI). For the 3 parameters of heterozygosity (Ho, He and UHe) of the 34 X-markers in the 5 ethnic groups, significant differences (p < 0.0001) were observed. Nei genetic distances between HUI and Uygur are always the highest, regardless of the markers used for analysis. HAN, Tibetan and Mongolian ethnic groups were closely clustered when analyzed with the 34 X-Chromosome markers. Although Indels are less polymorphic compared with STRs, they provided in general congruent phylogenetic signal and same cluster among the 5 ethnic groups. These results demonstrated that geographic isolation and interactions play significant roles in differentiation of genetic constitution of ethnic groups. However, the observed genetic relationships could be complicated by many factors including the origins of samples, the choice of genetic markers and coverage of reference populations. Thus, great care must be taken when relating one Chinese ethnic group to another, as there may have been intermixture with now formally extinct populations that contributes to current population genetic structure.

## Methods

Human blood samples were collected upon approval of Ethics Committee at the Institute of Forensic Sciences, Ministry of Justice, P. R. China. A written informed consent was obtained for each participant in this study. The main experiments were conducted at the Forensic Genetics Laboratory of Institute of Forensic Science, Ministry of Justice, P.R. China, which is an accredited laboratory by ISO 17025, in accordance with quality control measures. All the methods were carried out in accordance with the approved guidelines of Institute of Forensic Sciences, Ministry of Justice, P.R. China.

### Sample collection and DNA extraction

In this study, peripheral blood samples of healthy unrelated individuals from 5 main ethnic groups (HAN, HUI, Uygur, Mongolian and Tibetan) of China were collected. The detailed location information of the 5 ethnic groups is labeled in Supplementary Fig. S1. For each ethnic group, 100 individuals (50 females and 50 males) were included for study. Genomic DNA was extracted with QIAamp DNA Blood Mini Kit (QIAGEN, Hilden, Germany). DNA concentration was determined by the Quantifiler Human DNA Quantification Kit (Applied Biosystems, Foster City, CA) with 7500 Real-time PCR System (Applied Biosystems, Foster City, CA).

### Markers and genotyping

34 X-Chromosome markers for this study here were inferred to 18 X-InDels and 16 X-STRs. Detailed information of the amplification system is listed in the Supplementary Table S1. The 16 X-STRs (DXS10134, DXS10159, DXS6789, DXS6795, DXS6800, DXS6803, DXS6807, DXS6810, DXS7132, DXS7424, DXS8378, DXS9902, GATA165B12, GATA172D05, GATA31E08, HPRTB) were amplified with the in-house developed Panel I, whereas the 18 X-InDels (rs3048996, rs5901519, rs363794, rs2308033, rs66676381, rs5903978, rs3080039, rs55877732, rs10699224, rs2308280, rs45449991, rs3215490, rs25581, rs60283667, rs35574346, rs3047852, rs57608175, rs72417152) were amplified with the in-house developed Panel II. The PCR conditions for these two different types of markers were the same. The primer information is listed in Table 3.

PCR products were then analyzed by mixing 1 μL of each amplified product, with 9 μL of a 18:1 mixture of Hi-Di formamide (Applied Biosystem, Foster City, CA) and SIZ 500 (AGCU Co, China) for capillary electrophoresis. Electrophoresis was performed on AB 3130*xl* Genetic Analyzer (Applied Biosystems, Foster City, CA). Genotyping data were analyzed with GeneMapper v3.2.1 software (Applied Biosystems, Foster City, CA). Control DNA of 9947A (Applied Biosystem, Foster City, CA) was used as positive sample.

### Analytical method

Analyses of genetic parameters including allelic frequencies, observed and expected heterozygosities, Hardy-Weinberg equilibrium testing were performed with GENALEX 6.3. Exact tests for differentiation between female and male were performed with Arlequin. LD analysis was performed using the SNP Analyzer v2.0^22^. Population comparisons by mean of the Nei genetic distance and corresponding pairwise F_ST_ values, were assessed using GENALEX 6.3 software. For an easier visualization of the observed genetic distances, PCA analysis was reformed with GENALEX 6.3 software also. The significance level was based on 999 random permutations. Phylogenetic tree based on genetic distances was conducted with MEGA6 software using the Unweighted Pair Group Method with Arithmetic mean (UPGMA) method. Phylograms were created for STR and Indel marker data separately and also for combined datasets. The reliability of phylograms was estimated by bootstrapping 2000 replicates over loci and the extended majority rule consensus trees were inferred.

## Additional Information

**How to cite this article**: Zhang, S. *et al.* Population genetic study of 34 X-Chromosome markers in 5 main ethnic groups of China. *Sci. Rep.* **5**, 17711; doi: 10.1038/srep17711 (2015).

## Supplementary Material

Supplementary Information

## Figures and Tables

**Table 1:** Information of detected private alleles of the 16 X-STRs among the populations (N = 100 for each population, 50 females and 50 males).

Ethnic Group	STR	Private Allele	Detected Number	Allelic Frequency
SHANGHAI HAN	GATA165B12	13	3	0.020
SHANGHAI HAN	DXS8378	8	3	0.020
SHANGHAI HAN	DXS6803	14	3	0.020
SHANGHAI HAN	DXS7132	17	3	0.020
SHANGHAI HAN	DXS7132	18	3	0.020
Uygur	DXS101	17	12	0.080
Uygur	DXS101	19	6	0.040
Uygur	DX6809	28	12	0.080
Uygur	DXS7423	13	12	0.080
Mongolian	DXS981	18	6	0.040
Mongolian	DXS7423	17	6	0.040
Mongolian	DXS7133	19	12	0.080
Mongolian	DXS6810	10	12	0.080
Tibetan	GATA172D05	14	3	0.020
Tibetan	GATA172D05	15	3	0.020
Tibetan	HPRTB	10	6	0.040
Tibetan	DXS6795	10	6	0.040
Tibetan	DXS6795	11	6	0.040
Tibetan	GATA31E08	14	3	0.020
Tibetan	GATA31E08	15	3	0.020
Tibetan	GATA31E08	16	3	0.020
Tibetan	DXS6807	10	9	0.060
Tibetan	DXS7423	11	6	0.040

**Table 2:** Nei genetic distances among different pairwise ethnic groups with different kinds of markers.

Pairwise population	Nei genetic distances		
	A group (16 X-STRs)	B group (18 X-Indels)	C group (16 X-STRs+18 X-Indels)
HAN-HUI	0.045	0.035	0.065
HAN-Uygur	0.051	0.047	0.065
HAN-Mongolian	0.030	0.022	0.030
HAN-Tibetan	0.030	0.022	0.033
HUI-Uygur	0.095	0.075	0.125
HUI-Mongolian	0.047	0.028	0.087
HUI-Tibetan	0.051	0.032	0.065
Uygur-Mongolian	0.077	0.068	0.097
Uygur-Tibetan	0.050	0.037	0.079
Mongolian-Tibetan	0.022	0.012	0.052

**Table 3:** Primer information of Panel I (16 X-STRs) and Panel II (17 X-Indels).

X-STR	Primers (5′-3′)	X-InDel	Panel name	Primer sequence:(5′-3′)
DXS6795	F:CAAGGAGTTATGTTTTTAACTTAA	rs3048996	XF02	F: TGAATATACTGCAGGGTTCTGTTACAAC
	R:ACTGGGAGAAAAATCTCTTCAGTG			R: AGATAGACAGGAGATGAGTGAATGGCT
DXS9902	F:GTACTGTTCTTGTAACTTTTCTGTCG	rs55877732	XH03	F: ACCCTAGTTCTACAAAGCGCAAGTC
	R:CAGGAGTATGGGATCACCAGTGA			R: AAACATTTTTCAAGGGCAATGATGT
DXS8378	F:AGGAGGTTTGACCTGTTTCATTTCC	rs25581	XT06	F: CCTTTCTTGCACATTCTAGCTGAAC
	R:CTGAACTCCAGCCTGGGCGAC			R: GGGGTGGTGACAGAAGGGAAT
HPRTB	F:TCCATCTTTGTCTCTATCTCTATCT	rs10699224	XH08	F: TGTCACCACATTTCCTTCTGGGTA
	R:AAAAGCCATGGGAAAGAACTAGAAAG			R: TCACTTCCATTTGGCTTACTTCCTC
DXS6810	F:GTCATTAACCATATCTACATCTCTAC	rs5901519	XF09	F: TTGACGGGAATTGAGTCACCTG
	R:TAATTTTGTATTTTTAATAGAGACGGG			R: CTAAGGACAGCCTGAATCCCAGAT
GATA165B12	F:ATAAATTCTGAATAATCTATGTAT	rs60283667	XT12	F: CCTTATTTTGTGCCTTTTATTCTTGG
	R:GTGATTCCTGGTTTATATCTATTT			R: TCCTCTAAATTGGGGACCTATGTGTA
DXS7132	F:CTTAGCCTCCTTAATAGTGTGAGC	rs2308280	XH13	F: CTACCAACAAAATCCATTCTGGAATAA
	R:TTAGTCAACGTTCTCCAGAGAA			R: GTTTTATAGCAACACAAAATTGACTAAGACA
DXS7424	F:AGCTGAGGTGGGAGGACTGCT	rs35574346	XT14	F: ACTGGTAGGATCTGGAACATCTGC
	R:AGAGCAAAGACCCAGGCTGGTCCAGG			R: TGACTGTGGGCTTAAATCAAAACTT
DXS6807	F:AAAATACTCCCACCCCCAGTGGATGC	rs3047852	XT15	F: TTCCCAGATTTGAAATGTATGAAACTCT
	R:CGTAATATATGAAAAGAAAGGAAGC			R: CCTTAGTGCCTTGTTAGAAGGAATGA
DXS6803	F:ATGTGCTTTGACAGGAAAAACAATA	rs45449991	XH22	F: CCGAGTAGAGCTTAACATTTATACCTG
	R:ACAAAAAGGAACATATGCTACTTTATC			R: AGACATGATTGTGCCACTGGATTT
GATA172D05	F:GATAGTGGTGATGGTTGCACAGATATA	rs3215490	XH26	F: TCATCTATATTGAGTCAGCATTTGAACC
	R:TGATCCAGCAATTCTCCTTCTGGG			R: CAGAATCTCTGGAAACACTTGGTAGAA
DXS6800	F:AGGGCCTATTGTGGGACCTTGTGA	rs363794	XF28	F: TGGTCTCTGGAGTGCAATTTTAAGTC
	R:TTAGGGAAAATAGAAAAGAACCTACG			R: CCAAGCCAGCCATTTGTTCTTC
DXS10134	F:CACTGCCCTGCACTCCAGCC	rs57608175	XT32	F: CAAATTGACTATAGCCTTCCACCCT
	R:CCCCTGCTTTCAATTCTTTCTTTCTTTC			R: TGCCTTCCTCTCATTGACTTCATAA
GATA31E08	F:GATAGATGATAGAGATAGATGATAG	rs72417152	XT36	F: TGTAAAGCTACACCAATGGACAGATG
	R:TGCTCACTTTTATGTGTGTATGTATC			R: TGCAAAGATTAAGTGCATTTTCTCTG
DXS10159	F:CATAAGCGAAACTCTATCTCAG	rs2308033	XF37	F: ATGGCATTTAGTCTCAGGTCTGCTTA
	R:TTTTGACATGTTGCTGGATTTGG			R: CAACTGGTCCACCCTAACTGTATCC
DXS6789	F:TATTGTGGGACCTCGTGATCATG	rs66676381	XF38	F: CTTTGGGTGATAGGAGGTTTTGC
	R:GGCAAAAAAATTAGGAGATCTAAGAAG			R: AACCCCTGGTGCTGTGTGTAAAT
		rs5903978	XF39	F: TGTACCACTGTAAAGCCCCCG
				R: GCTGCATTGTCTGGCAATGAA
		rs3080039	XF46	F: ACCTTGGACAAAGTTACTTAGCTGCT
				R: TAGCAAGTCACACATGGATGAGAAA
