# Supplementary material for: Population genetic study of 34 X-Chromosome markers in 5 main ethnic groups of China
Source: Sci Rep. 2015 Dec 4;5:17711. doi: 10.1038/srep17711 (PMC4669481; doi:10.1038/srep17711)
Supplement: Supplementary Information [file srep17711-s1.pdf]

Supplementary Figures S1-S4 and Supplementary Tables S1-S3

Population genetic study of 34 X-Chromosome markers in 5 main ethnic groups of China

Suhua Zhang[1,2], Yingnan Bian[1], Li Li[1], Kuan Sun[3], Zheng Wang[1], Qi Zhao[1], Lagabaiyila Zha[4], Jifeng Cai[4], Yuzhen Gao[5], Chaoneng Ji[2], Chengtao Li[1]*

[1] Shanghai Key Laboratory of Forensic Medicine, Institute of Forensic Sciences, Ministry of Justice, P.R. China, Shanghai 200063, P.R. China

[2] State Key Laboratory of Genetic Engineering, Institute of Genetics, School of Life Sciences, Fudan University, Shanghai 200433, P.R. China

[3] Institute of Forensic Medicine, West China School of Basic Science and Forensic Medicine, Sichuan University, Chengdu 610041, P.R.China

[4] Department of Forensic Science, School of Basic Medical Sciences, Central South University, Changsha 410013, P.R. China

[5] Department of Forensic Medicine, Medical College of Soochow University, Suzhou 215123, P.R. China

***** Corresponding author: Chengtao Li.

Shanghai Key Laboratory of Forensic Medicine, Institute of Forensic Sciences, Ministry of Justice, P.R. China, Shanghai 200063, P.R. China

E-mail: lichengtaohla@163.com; Phone: +86-21-52351327; Fax: +86-21-52352959.

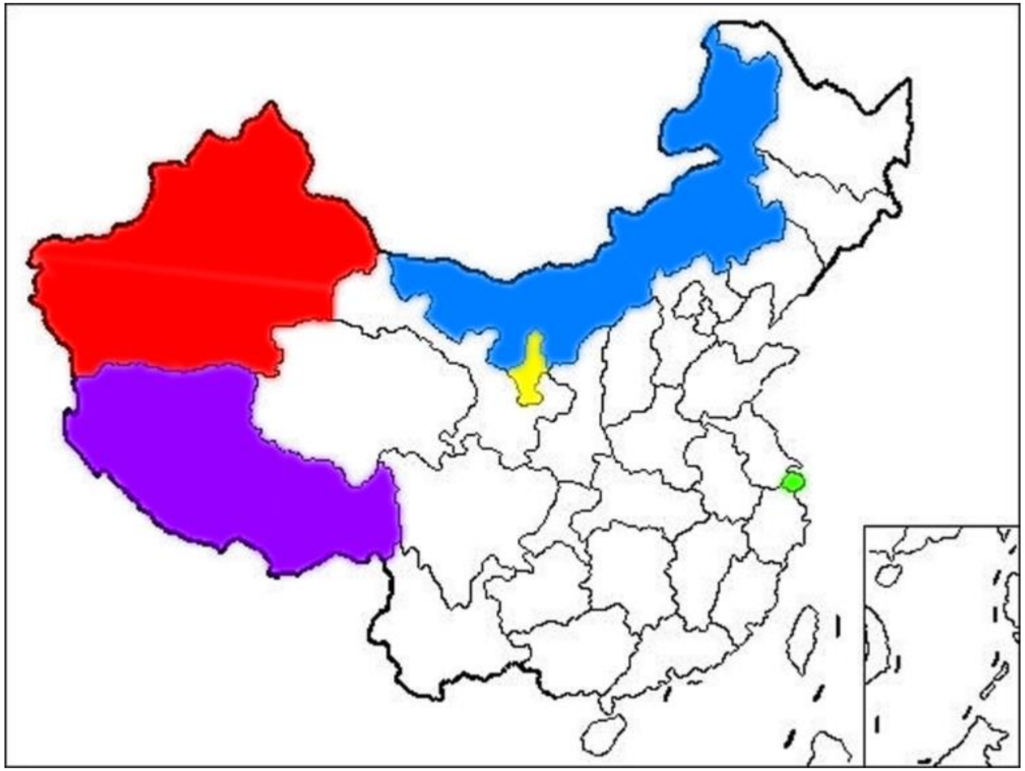

Supplementary Fig S1. Detailed location of the 5 studied ethnic groups (HAN, HUI, Uygur, Mongolian and Tibetan) in China and color labeling. *Green:* HAN population in Shanghai, *yellow:* HUI population in Ningxia, *red:* Uygur population in Xinjiang, *blue:* Mongolian population in Inner Mongolian, *purple:* Tibetan population in Xizang. The map was created by software of R 3.1.2.

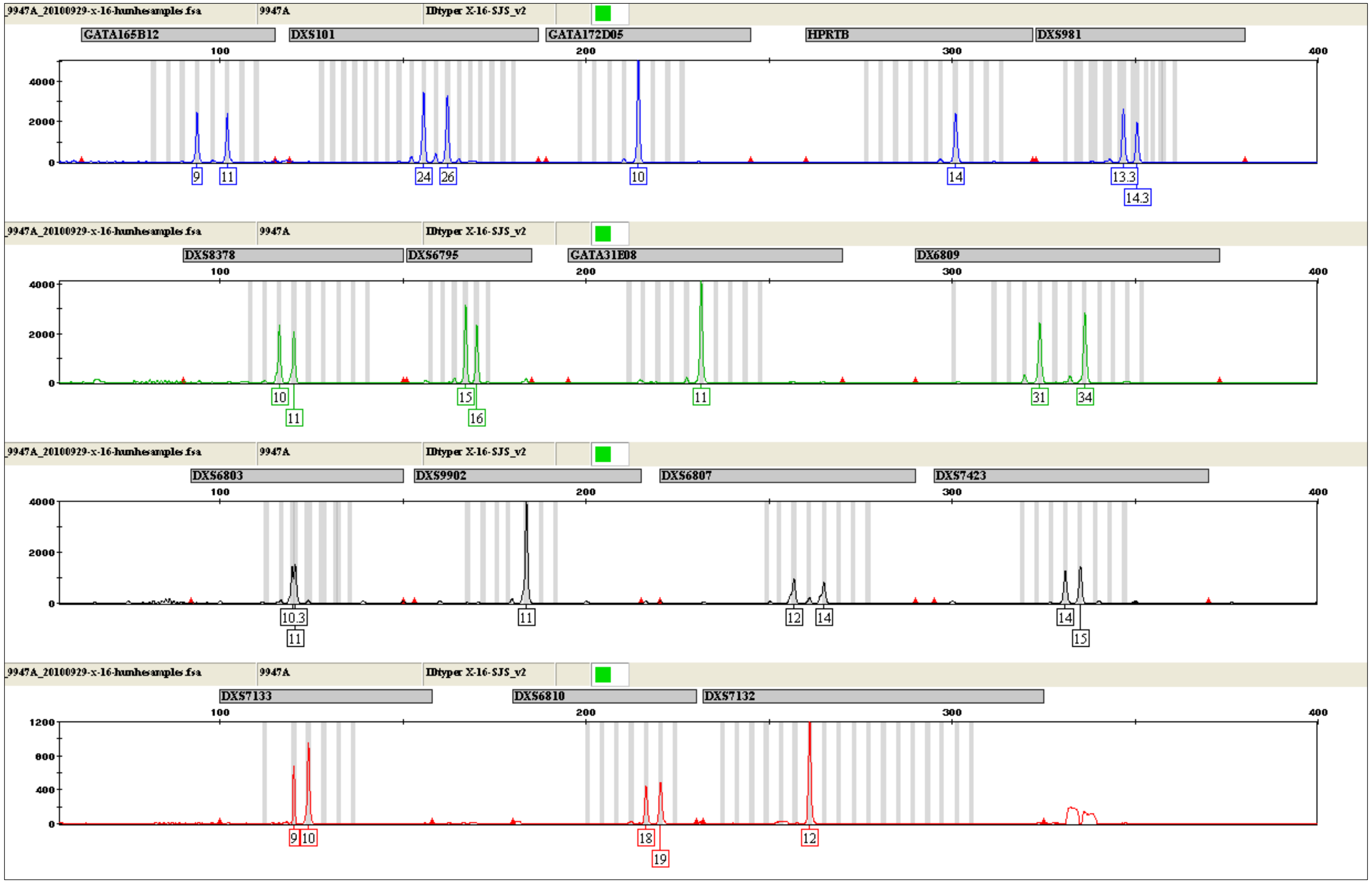

Supplementary Fig S2-A. Genotyping profile of control DNA 9947A (0.5 ng) amplified with Panel I.

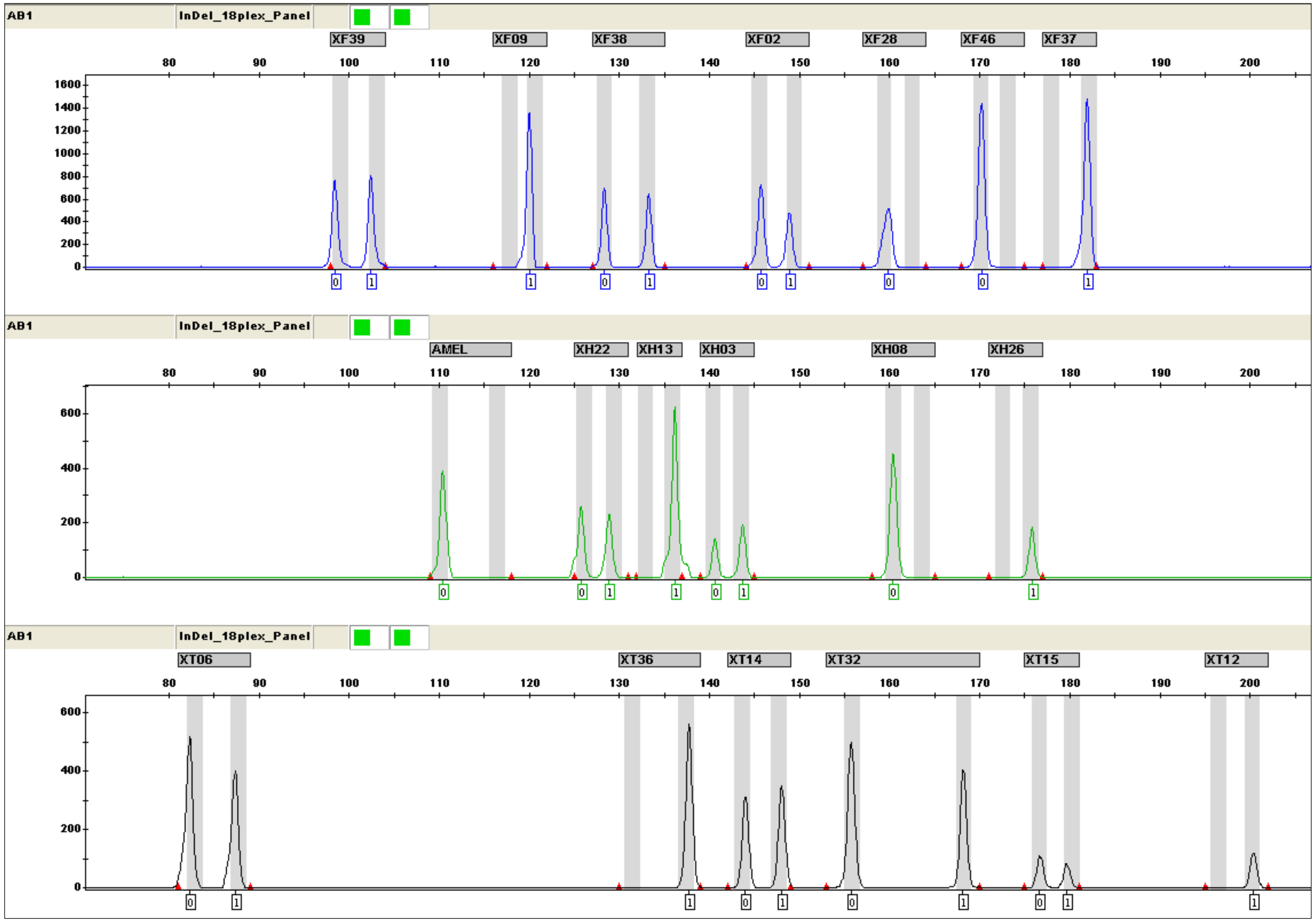

Supplementary Fig S2-B. Genotyping profile of control DNA 9947A (0.5 ng) amplified with Panel II.

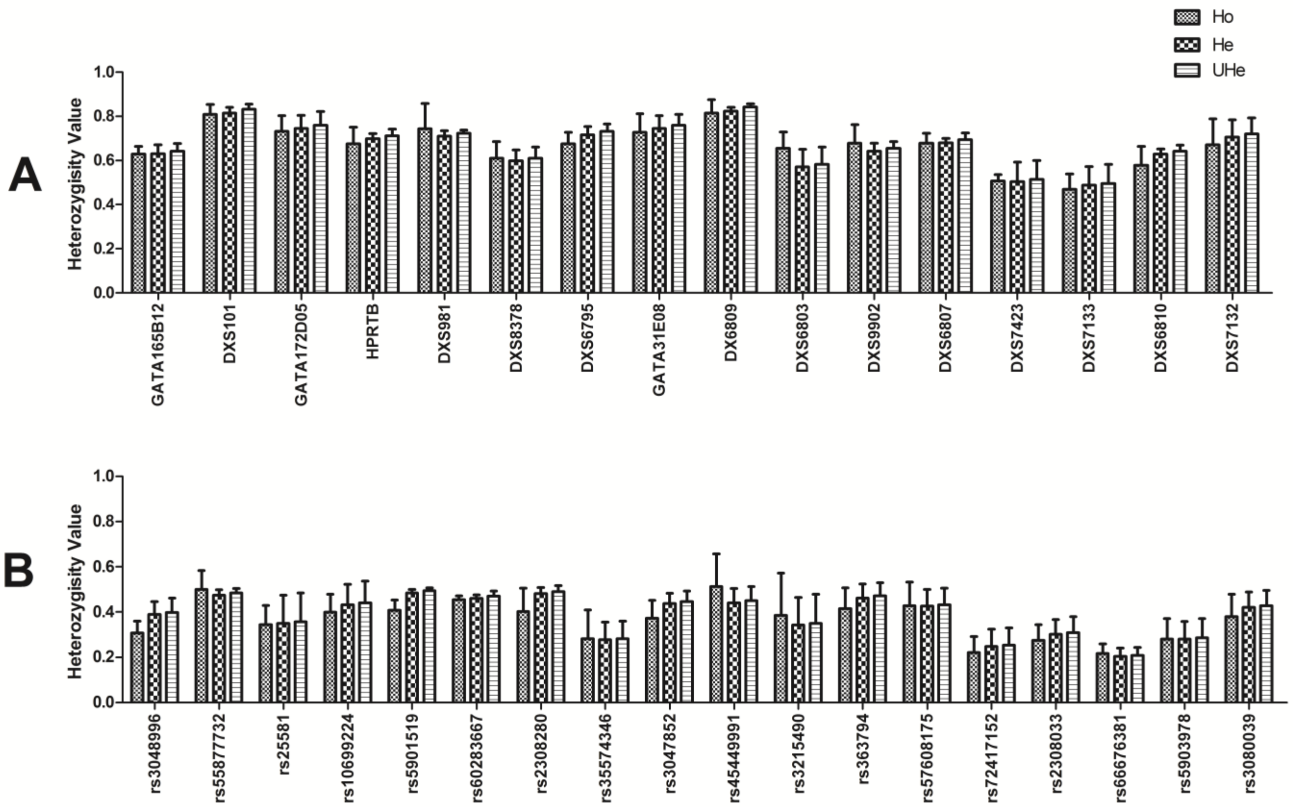

Supplementary Fig S3. Heterozygosity values (mean±SD) of X-Chromosome markers for the 5 ethnic groups. A: 16 X-Chromosome STRs; B: 18 X-Chromosome Indels.

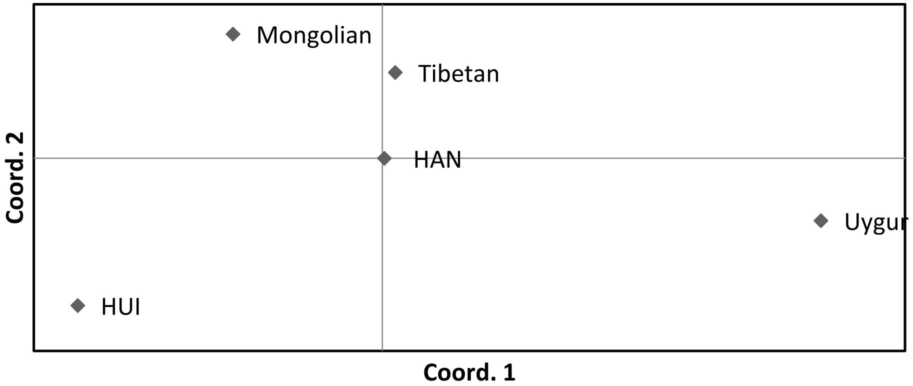

Supplementary Fig S4-A. PCA based on Nei genetic distance matrix analyzed with 16 X-STRs among the 5 ethnic groups

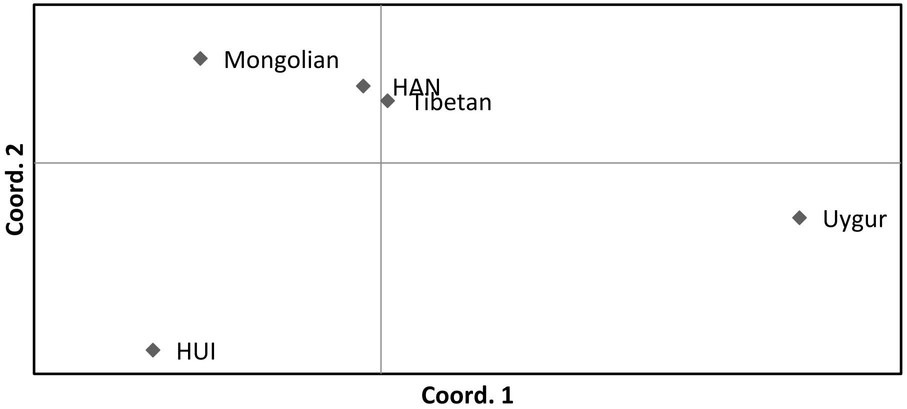

Supplementary Fig S4-B. PCA based on Nei genetic distance matrix analyzed with 18 X-Indels among the 5 ethnic groups

Supplementary Table S1. Amplification system and cycling conditions of Panel I and II

**A. Reaction component**	Panel I	Panel II
1× Qiagen multiplex PCR master mix	5	5
1×Q-Solution	2.5	2.5
primer mix	2 (Panel I primer mix)	2 (Panel II primer mix)
gDNA (0.5-2 ng)	1	1
H2O	2	2
**B. Thermal cycling condition**		
Initial active	95°C, 15min	
	94°C, 30s	
PCR cycles (30)	65°C, 90s	
	72°C, 90s	
Extension step	60°C, 60min	

Supplementary Table S2. Allelic frequencies of the 34 X-Chromosomal markers in 5 main ethnic groups of China (N=100 for each population, 50 females and 50 males).

Loci	Allele	HAN	HUI	Uygur	Mongolian	Tibetan	Loci	Allele	HAN	HUI	Uygur	Mongolian	Tibetan
rs3048996	D	0.1875	0.3636	0.2800	0.2679	0.2604	DXS8378	8	0.008	0.000	0.000	0.000	0.000
	I	0.8125	0.6364	0.7200	0.7321	0.7396		9	0.016	0.000	0.020	0.000	0.021
rs55877732	D	0.5000	0.3636	0.3400	0.3571	0.5104		10	0.563	0.500	0.520	0.589	0.417
	I	0.5000	0.6364	0.6600	0.6429	0.4896		11	0.289	0.364	0.300	0.357	0.385
rs25581	D	0.8906	0.7727	0.5000	0.8393	0.6771		12	0.109	0.136	0.160	0.036	0.094
	I	0.1094	0.2273	0.5000	0.1607	0.3229		13	0.008	0.000	0.000	0.018	0.073
rs10699224	D	0.3125	0.4091	0.0400	0.6071	0.4479		14	0.008	0.000	0.000	0.000	0.010
	I	0.6875	0.5909	0.9600	0.3929	0.5521	DXS6795	10	0.000	0.000	0.000	0.000	0.021
rs5901519	D	0.5703	0.3636	0.3800	0.5000	0.5208		11	0.000	0.000	0.000	0.000	0.021
	I	0.4297	0.6364	0.6200	0.5000	0.4792		12	0.016	0.045	0.180	0.036	0.063
rs60283667	D	0.3438	0.5909	0.3400	0.3750	0.3438		13	0.203	0.364	0.160	0.179	0.208
	I	0.6563	0.4091	0.6600	0.6250	0.6563		14	0.297	0.136	0.360	0.250	0.240
rs2308280	D	0.5000	0.4091	0.3200	0.5357	0.4271		15	0.031	0.045	0.020	0.054	0.104
	I	0.5000	0.5909	0.6800	0.4643	0.5729		16	0.422	0.409	0.260	0.446	0.344
rs35574346	D	0.2656	0.0455	0.1000	0.0893	0.0938		17	0.031	0.000	0.020	0.036	0.000
	I	0.7344	0.9546	0.9000	0.9107	0.9063	GATA31E08	6	0.000	0.000	0.000	0.000	0.010
rs3047852	D	0.3750	0.3182	0.4600	0.2679	0.2708		7	0.055	0.000	0.120	0.036	0.073
	I	0.6250	0.6818	0.5400	0.7321	0.7292		8	0.055	0.000	0.020	0.054	0.063
rs45449991	D	0.5313	0.7273	0.5400	0.6429	0.7708		9	0.188	0.136	0.180	0.232	0.177
	I	0.4688	0.2727	0.4600	0.3571	0.2292		10	0.227	0.182	0.340	0.196	0.198
rs3215490	D	0.3750	0.4091	0.1400	0.1607	0.1458		11	0.344	0.500	0.220	0.446	0.333

Loci	Allele	HAN	HUI	Uygur	Mongolian	Tibetan	Loci	Allele	HAN	HUI	Uygur	Mongolian	Tibetan
	I	0.6250	0.5909	0.8600	0.8393	0.8542		12	0.117	0.182	0.100	0.036	0.083
rs363794	D	0.4453	0.2273	0.6200	0.4464	0.5000		13	0.016	0.000	0.020	0.000	0.031
	I	0.5547	0.7727	0.3800	0.5536	0.5000		14	0.000	0.000	0.000	0.000	0.010
rs57608175	D	0.6719	0.8182	0.4800	0.6786	0.6563		15	0.000	0.000	0.000	0.000	0.010
	I	0.3281	0.1818	0.5200	0.3214	0.3438		16	0.000	0.000	0.000	0.000	0.010
rs72417152	D	0.1875	0.0909	0.2200	0.1429	0.1042	DX6809	28	0.000	0.000	0.040	0.000	0.000
	I	0.8125	0.9091	0.7800	0.8571	0.8958		29	0.016	0.000	0.020	0.018	0.000
rs2308033	D	0.1719	0.2727	0.1400	0.2143	0.1458		30	0.023	0.000	0.060	0.036	0.031
	I	0.8281	0.7273	0.8600	0.7857	0.8542		31	0.180	0.136	0.140	0.143	0.188
rs66676381	D	0.8672	0.9091	0.8600	0.9107	0.8750		32	0.117	0.136	0.120	0.179	0.156
	I	0.1328	0.0909	0.1400	0.0893	0.1250		33	0.227	0.182	0.320	0.232	0.198
rs5903978	D	0.7969	0.7273	0.9400	0.8750	0.8542		34	0.242	0.273	0.140	0.143	0.292
	I	0.2031	0.2727	0.0600	0.1250	0.1458		35	0.117	0.227	0.100	0.089	0.115
rs3080039	D	0.6797	0.7273	0.6000	0.8036	0.6146		36	0.070	0.045	0.020	0.125	0.010
	I	0.3203	0.2727	0.4000	0.1964	0.3854		37	0.008	0.000	0.020	0.018	0.010
GATA165B12	8	0.0000	0.0000	0.0200	0.0000	0.0104		38	0.000	0.000	0.020	0.018	0.000
	9	0.2734	0.1364	0.3600	0.1250	0.1979	DXS6803	9	0.008	0.045	0.020	0.000	0.021
	10	0.5000	0.5909	0.4400	0.5536	0.4375		10	0.148	0.273	0.340	0.054	0.156
	11	0.1484	0.2273	0.1800	0.2857	0.2917		11	0.203	0.045	0.360	0.250	0.208
	12	0.0703	0.0455	0.0000	0.0357	0.0625		12	0.594	0.636	0.260	0.679	0.594
	13	0.0078	0.0000	0.0000	0.0000	0.0000		13	0.039	0.000	0.020	0.018	0.021
DXS101	14	0.000	0.000	0.020	0.000	0.000		14	0.008	0.000	0.000	0.000	0.000
	17	0.000	0.000	0.040	0.000	0.000	DXS9902	9	0.039	0.000	0.020	0.000	0.031
	18	0.000	0.000	0.020	0.000	0.000		10	0.391	0.545	0.220	0.411	0.510
	19	0.000	0.000	0.020	0.000	0.000		11	0.359	0.318	0.440	0.268	0.281

Loci	Allele	HAN	HUI	Uygur	Mongolian	Tibetan	Loci	Allele	HAN	HUI	Uygur	Mongolian	Tibetan
	20	0.016	0.045	0.020	0.018	0.021		12	0.211	0.136	0.320	0.304	0.167
	21	0.016	0.000	0.040	0.054	0.010		13	0.000	0.000	0.000	0.018	0.010
	22	0.039	0.000	0.020	0.000	0.000	DXS6807	10	0.000	0.000	0.000	0.000	0.031
	23	0.172	0.091	0.060	0.214	0.104		11	0.453	0.409	0.440	0.446	0.375
	24	0.320	0.273	0.140	0.143	0.229		12	0.016	0.045	0.020	0.018	0.031
	25	0.203	0.227	0.260	0.250	0.260		13	0.031	0.045	0.040	0.089	0.000
	26	0.148	0.273	0.200	0.250	0.188		14	0.359	0.318	0.320	0.250	0.396
	27	0.047	0.045	0.120	0.054	0.083		15	0.094	0.136	0.180	0.196	0.156
	28	0.008	0.000	0.020	0.018	0.052		16	0.047	0.045	0.000	0.000	0.010
	29	0.000	0.045	0.020	0.000	0.000	DXS7423	11	0.000	0.000	0.000	0.000	0.021
	30	0.016	0.000	0.000	0.000	0.010		13	0.000	0.000	0.040	0.000	0.000
	31	0.008	0.000	0.000	0.000	0.042		14	0.352	0.273	0.300	0.268	0.323
	32	0.008	0.000	0.000	0.000	0.000		15	0.625	0.727	0.500	0.679	0.594
GATA172D05	6	0.094	0.000	0.120	0.125	0.073		16	0.023	0.000	0.160	0.036	0.063
	7	0.000	0.000	0.020	0.000	0.000		17	0.000	0.000	0.000	0.018	0.000
	8	0.133	0.273	0.180	0.071	0.031	DXS7133	6	0.008	0.000	0.000	0.000	0.021
	9	0.172	0.091	0.100	0.161	0.083		9	0.719	0.955	0.580	0.643	0.708
	10	0.375	0.364	0.280	0.411	0.531		10	0.219	0.045	0.180	0.179	0.188
	11	0.180	0.227	0.200	0.161	0.229		11	0.055	0.000	0.160	0.036	0.063
	12	0.047	0.045	0.100	0.071	0.010		12	0.000	0.000	0.040	0.000	0.000
	14	0.000	0.000	0.000	0.000	0.010		17	0.000	0.000	0.020	0.018	0.000
	15	0.000	0.000	0.000	0.000	0.031		18	0.000	0.000	0.020	0.089	0.021
HPRTB	10	0.000	0.000	0.000	0.000	0.021		19	0.000	0.000	0.000	0.036	0.000
	11	0.031	0.045	0.020	0.018	0.063	DXS6810	9	0.000	0.000	0.040	0.107	0.021
	12	0.320	0.227	0.300	0.411	0.177		10	0.000	0.000	0.000	0.036	0.000

Loci	Allele	HAN	HUI	Uygur	Mongolian	Tibetan	Loci	Allele	HAN	HUI	Uygur	Mongolian	Tibetan
	13	0.391	0.227	0.420	0.375	0.385		16	0.008	0.000	0.000	0.000	0.010
	14	0.180	0.409	0.160	0.179	0.333		17	0.148	0.227	0.160	0.036	0.125
	15	0.078	0.091	0.080	0.018	0.021		18	0.531	0.500	0.480	0.500	0.500
	16	0.000	0.000	0.020	0.000	0.000		19	0.313	0.273	0.280	0.321	0.333
DXS981	12	0.117	0.182	0.120	0.107	0.167		20	0.000	0.000	0.040	0.000	0.010
	13	0.297	0.182	0.340	0.411	0.375	DXS7132	11	0.000	0.000	0.020	0.018	0.010
	14	0.375	0.500	0.380	0.250	0.292		12	0.094	0.045	0.120	0.107	0.094
	15	0.172	0.136	0.100	0.214	0.135		13	0.227	0.091	0.220	0.214	0.292
	16	0.039	0.000	0.060	0.000	0.031		14	0.359	0.591	0.380	0.232	0.385
	18	0.000	0.000	0.000	0.018	0.000		15	0.250	0.273	0.180	0.375	0.198
								16	0.055	0.000	0.080	0.054	0.021
								17	0.008	0.000	0.000	0.000	0.000
								18	0.008	0.000	0.000	0.000	0.000

D: Deletion; I: Insertion.

Supplementary Table S3. Heterozygosity information of the 34 X-Chromosomal markers among the 5 ethnic groups

Marker	HAN			HUI			Uygur			Mongolian			Tibetan		
	Ho	He	UHe	Ho	He	UHe	Ho	He	UHe	Ho	He	UHe	Ho	He	UHe
rs3048996	0.2813	0.3047	0.3071	0.3818	0.4628	0.4848	0.2400	0.4032	0.4114	0.3214	0.3922	0.3994	0.3125	0.3852	0.3893
rs55877732	0.5625	0.5000	0.5039	0.3636	0.4628	0.4848	0.5200	0.4488	0.4580	0.5714	0.4592	0.4675	0.4792	0.4998	0.5050
rs25581	0.2188	0.1948	0.1964	0.3509	0.3512	0.3680	0.4400	0.5000	0.5102	0.3214	0.2698	0.2747	0.3958	0.4373	0.4419
rs10699224	0.4688	0.4297	0.4331	0.4545	0.4835	0.5065	0.2800	0.2768	0.2784	0.3571	0.4770	0.4857	0.4375	0.4946	0.4998
rs5901519	0.4219	0.4901	0.4940	0.3636	0.4628	0.4848	0.4400	0.4712	0.4808	0.3571	0.5000	0.5091	0.4583	0.4991	0.5044
rs60283667	0.4375	0.4512	0.4547	0.4545	0.4835	0.5065	0.4400	0.4488	0.4580	0.4643	0.4688	0.4773	0.4792	0.4512	0.4559
rs2308280	0.4688	0.5000	0.5039	0.2727	0.4835	0.5065	0.3200	0.4352	0.4441	0.4286	0.4974	0.5065	0.5208	0.4894	0.4945
rs35574346	0.5000	0.3901	0.3932	0.2909	0.2868	0.2909	0.2200	0.2800	0.2837	0.2071	0.2626	0.2656	0.1875	0.1699	0.1717
rs3047852	0.5000	0.4688	0.4724	0.3909	0.4339	0.4545	0.3600	0.4968	0.5069	0.3214	0.3922	0.3994	0.2917	0.3950	0.3991
rs45449991	0.7500	0.4980	0.5020	0.3818	0.3967	0.4156	0.5200	0.4968	0.5069	0.5000	0.4592	0.4675	0.4167	0.3533	0.3570
rs3215490	0.5313	0.4688	0.4724	0.6364	0.4835	0.5065	0.2400	0.2408	0.2457	0.2357	0.2698	0.2747	0.2833	0.2491	0.2518
rs363794	0.5156	0.4940	0.4979	0.2727	0.3512	0.3680	0.4400	0.4712	0.4808	0.3929	0.4943	0.5032	0.4583	0.5000	0.5053
rs57608175	0.5938	0.4409	0.4444	0.3636	0.2975	0.3117	0.3200	0.4992	0.5094	0.4286	0.4362	0.4442	0.4375	0.4512	0.4559
rs72417152	0.3438	0.3047	0.3071	0.1818	0.1653	0.1732	0.2000	0.3432	0.3502	0.2143	0.2449	0.2494	0.1667	0.1866	0.1886
rs2308033	0.2813	0.2847	0.2869	0.3818	0.3967	0.4156	0.2200	0.2408	0.2457	0.2857	0.3367	0.3429	0.2083	0.2491	0.2518
rs66676381	0.2344	0.2303	0.2322	0.1818	0.1653	0.1732	0.2800	0.2408	0.2457	0.1786	0.1626	0.1656	0.2083	0.2188	0.2211
rs5903978	0.3875	0.3237	0.3263	0.3636	0.3967	0.4156	0.2200	0.2128	0.2151	0.1786	0.2188	0.2227	0.2500	0.2491	0.2518
rs3080039	0.3594	0.4354	0.4389	0.3818	0.3967	0.4156	0.2400	0.4800	0.4898	0.3929	0.3157	0.3214	0.5208	0.4737	0.4787
GATA165B12	0.6250	0.6482	0.6533	0.6364	0.5785	0.6061	0.6400	0.6440	0.6571	0.5714	0.5950	0.6058	0.6667	0.6803	0.6875
DXS101	0.8594	0.7999	0.8062	0.7832	0.7851	0.8225	0.8400	0.8488	0.8661	0.7500	0.8023	0.8169	0.8125	0.8314	0.8401
GATA172D05	0.7500	0.7689	0.7750	0.6364	0.7314	0.7662	0.7600	0.8144	0.8310	0.8214	0.7538	0.7675	0.6875	0.6515	0.6583
HPRTB	0.6094	0.7054	0.7110	0.6364	0.7190	0.7532	0.8000	0.7008	0.7151	0.6429	0.6582	0.6701	0.6875	0.7042	0.7116

Marker	HAN			HUI			Uygur			Mongolian			Tibetan		
	**Ho**	**He**	**UHe**	**Ho**	**He**	**UHe**	**Ho**	**He**	**UHe**	**Ho**	**He**	**UHe**	**Ho**	**He**	**UHe**
DXS981	0.8438	0.7264	0.7322	0.6364	0.6653	0.6970	0.6000	0.7120	0.7265	0.8214	0.7111	0.7240	0.8125	0.7272	0.7349
DXS8378	0.5625	0.5876	0.5923	0.5455	0.5992	0.6277	0.6400	0.6136	0.6261	0.5714	0.5236	0.5331	0.7292	0.6632	0.6702
DXS6795	0.7500	0.6904	0.6959	0.6364	0.6777	0.7100	0.6400	0.7440	0.7592	0.6429	0.7009	0.7136	0.7083	0.7654	0.7735
GATA31E08	0.6875	0.7754	0.7815	0.6364	0.6653	0.6970	0.8400	0.7784	0.7943	0.6786	0.7028	0.7156	0.7917	0.8008	0.8092
DX6809	0.7969	0.8245	0.8310	0.7273	0.8017	0.8398	0.8400	0.8272	0.8441	0.8929	0.8476	0.8630	0.8125	0.8181	0.8268
DXS6803	0.6719	0.5825	0.5871	0.6364	0.5165	0.5411	0.7200	0.6864	0.7004	0.5357	0.4739	0.4825	0.7083	0.5890	0.5952
DXS9902	0.6094	0.6722	0.6775	0.7273	0.5826	0.6104	0.8000	0.6552	0.6686	0.6071	0.6671	0.6792	0.6458	0.6315	0.6382
DXS6807	0.6406	0.6533	0.6585	0.7273	0.7066	0.7403	0.7200	0.6696	0.6833	0.6786	0.6913	0.7039	0.6250	0.6762	0.6833
DXS7423	0.5000	0.4852	0.4891	0.5455	0.3967	0.4156	0.5200	0.6328	0.6457	0.4643	0.4662	0.4747	0.5000	0.5388	0.5445
DXS7133	0.4375	0.4325	0.4359	0.4016	0.4028	0.4033	0.5600	0.6032	0.6155	0.5269	0.5440	0.5539	0.4167	0.4583	0.4632
DXS6810	0.5469	0.5980	0.6027	0.7273	0.6240	0.6537	0.5600	0.6624	0.6759	0.5357	0.6327	0.6442	0.5208	0.6226	0.6292
DXS7132	0.7656	0.7451	0.7510	0.6364	0.5661	0.5931	0.8000	0.7536	0.7690	0.5000	0.7449	0.7584	0.6458	0.7179	0.7254

Ho: Observed Heterozygosity; He: Expected Heterozygosity; UHe: Unbiased Expected Heterozygosity
